# Supplementary material for: Longitudinal patterns of digital parenting restrictions and adolescent screen use: Insights from the Adolescent Brain Cognitive Development (ABCD) study
Source: PLOS Digit Health. 2026 Oct 28;5(10):e0001127. doi: 10.1371/journal.pdig.0001127 (PMC13716224; doi:10.1371/journal.pdig.0001127)
Supplement: Supplementary file 1 [file pdig.0001127.s001.docx]

**S1 Table.** The Parent Screen Time Questionnaire

1. When I am with my child, I use a screen-based device.

2. I try to limit how much I use a screen-based device when I am with my child.

3. Our family often watches a screen during meals.

4. Family members are allowed to use screen-based devices during meals.

5. My child falls asleep while using a screen-based device.

6. A screen-based device is usually playing in the room when my child falls asleep.

7. My child has access to a mobile screen-based device in bed.

8. I offer screen time to my child as a reward for good behavior.

9. I take away screen time from my child as a punishment for bad behavior.

10. I keep track of my child's screen time during the week.

11. I keep track of my child's screen time during the weekend.

12. I limit my child's screen time during the week.

13. I limit my child's screen time during the weekend.

14. I encourage my child to do activities other than screen time.

**Possible responses:** 1=Strongly Disagree, 2=Somewhat Disagree, 3= Somewhat Agree, 4=Strongly Agree
