# Supplementary material for: Longitudinal patterns of digital parenting restrictions and adolescent screen use: Insights from the Adolescent Brain Cognitive Development (ABCD) study
Source: PLOS Digit Health. 2026 Oct 28;5(10):e0001127. doi: 10.1371/journal.pdig.0001127 (PMC13716224; doi:10.1371/journal.pdig.0001127)
Supplement: Supplementary file 2 [file pdig.0001127.s002.docx]

| **S2 Table.** Reliability Estimates (McDonald's Omega) for PSQ Items Across Years 3, 4, and 5 | | | | |
| --- | --- | --- | --- | --- |
|  | **McDonald's Omega (ω)** | | |  |
|  | **β** | **SE** | **p-value** |  |
| **Visit** |  |  |  |  |
| Year-3 | 0.918 | 0.001 | <0.001 |  |
| Year-4 | 0.929 | 0.001 | <0.001 |  |
| Year-5 | 0.933 | 0.001 | <0.001 |  |
