# Supplementary material for: Longitudinal patterns of digital parenting restrictions and adolescent screen use: Insights from the Adolescent Brain Cognitive Development (ABCD) study
Source: PLOS Digit Health. 2026 Oct 28;5(10):e0001127. doi: 10.1371/journal.pdig.0001127 (PMC13716224; doi:10.1371/journal.pdig.0001127)
Supplement: Supplementary file 3 [file pdig.0001127.s003.docx]

| **S3 Table.** Frequencies and Proportions of Longitudinal Parenting Restriction Trajectories Across Years 3, 4, and 5 | | | | |
| --- | --- | --- | --- | --- |
| **Year 3** | **Year 4** | **Year 5** | **Count** | **Proportion (%)** |
| **High** | **High** | **High** | **1,739.0** | **21.5%** |
| High | High | Moderate | 633.0 | 7.8% |
| High | High | Low | 230.0 | 2.8% |
| High | Moderate | High | 55.0 | 0.7% |
| High | Moderate | Moderate | 556.0 | 6.9% |
| High | Moderate | Low | 250.0 | 3.1% |
| High | Low | High | 30.0 | 0.4% |
| High | Low | Moderate | 67.0 | 0.8% |
| High | Low | Low | 236.0 | 2.9% |
| Moderate | High | High | 89.0 | 1.1% |
| Moderate | High | Moderate | 75.0 | 0.9% |
| Moderate | High | Low | 14.0 | 0.2% |
| Moderate | Moderate | High | 56.0 | 0.7% |
| **Moderate** | **Moderate** | **Moderate** | **1,443.0** | **17.8%** |
| Moderate | Moderate | Low | 693.0 | 8.6% |
| Moderate | Low | High | 22.0 | 0.3% |
| Moderate | Low | Moderate | 172.0 | 2.1% |
| Moderate | Low | Low | 565.0 | 7.0% |
| Low | High | High | 38.0 | 0.5% |
| Low | High | Moderate | 11.0 | 0.1% |
| Low | High | Low | 20.0 | 0.2% |
| Low | Moderate | High | 3.0 | 0.0% |
| Low | Moderate | Moderate | 80.0 | 1.0% |
| Low | Moderate | Low | 106.0 | 1.3% |
| Low | Low | High | 12.0 | 0.1% |
| Low | Low | Moderate | 89.0 | 1.1% |
| **Low** | **Low** | **Low** | **805.0** | **10.0%** |
| Note. Each row represents a unique longitudinal trajectory of parenting restriction profiles across Years 3, 4, and 5. High, Moderate, and Low refer to restriction levels assigned at each study wave. Counts and proportions indicate the number and percentage of participants following each trajectory pattern. | | | | |
