# Supplementary material for: Longitudinal patterns of digital parenting restrictions and adolescent screen use: Insights from the Adolescent Brain Cognitive Development (ABCD) study
Source: PLOS Digit Health. 2026 Oct 28;5(10):e0001127. doi: 10.1371/journal.pdig.0001127 (PMC13716224; doi:10.1371/journal.pdig.0001127)
Supplement: Supplementary file 4 [file pdig.0001127.s004.docx]

| **S4 Table.** Latent Transition Analysis Model Fit Indices for Year 3 to Year 5 (n=8089) | | | | | | | | | | | |  |
| --- | --- | --- | --- | --- | --- | --- | --- | --- | --- | --- | --- | --- |
| **Number of Classes** | **LL** | **BIC** | **SABIC** | **CAIC** | **Entropy** | **Smallest off-diagonal ALCPP** | **Average latent class posterior probabilities** | | | |  |  |
|  |  |  |  |  |  |  |  |  |  |  |  |  |
| 2 | -368100 | 737002 | 736719 | 736379 | 0.868 | 0.936 | 0.972, 0.936 | | | |  |  |
| 3 | -352149 | 705557 | 705112 | 704578 | 0.876 | 0.919 | 0.949, 0.919, 0.933 | | | |  |  |
| 4 | -343633 | 689030 | 688407 | 687658 | 0.886 | 0.923 | 0.923, 0.933, 0.944, 0.923 | | | |  |  |
| 5 | -339385 | 681064 | 680254 | 679280 | 0.875 | 0.881 | 0.881. 0.889, 0.937, 0.91, 0.993 | | | |  |  |
| 6 | -335460 | 673782 | 672772 | 671557 | 0.871 | 0.871 | 0.871, 0.894, 0.933, 0.904, 0.888, 0.900 | | | |  |  |
| Note. LL = Log-Likelihood, CAIC = Consistent Akaike Information Criterion, BIC = Bayesian Information Criterion, SABIC = Sample-Size Adjusted Bayesian Information Criterion, ALCPP = Average Latent Class Posterior Probability | | | | | | | | | | | |  |
|  |  |  |  |  |  |  |  |  |  |  |  |  |
