# Supplementary material for: Longitudinal patterns of digital parenting restrictions and adolescent screen use: Insights from the Adolescent Brain Cognitive Development (ABCD) study
Source: PLOS Digit Health. 2026 Oct 28;5(10):e0001127. doi: 10.1371/journal.pdig.0001127 (PMC13716224; doi:10.1371/journal.pdig.0001127)
Supplement: Supplementary file 5 [file pdig.0001127.s005.docx]

| **S5 Table.** Fit Statistics and Class Enumeration for Latent Class Analysis by Time Point | | | | | | |  |
| --- | --- | --- | --- | --- | --- | --- | --- |
| **Model** | **# of classes** | **LL** | **BIC** | **SABIC** | **CAIC** | **Entropy** |  |
| Year-3 visit (T1) (n=7954) | 1 | -128006 | 256390 | 256257 | 256097 | NA |  |
|  | 2 | -120389 | 241543 | 241273 | 240949 | 0.816 |  |
|  | 3 | -116047 | 233245 | 232838 | 232351 | **0.882** |  |
|  | 4 | -113355 | 228247 | 227703 | 227053 | 0.881 |  |
|  | 5 | -112144 | 226211 | 225531 | 224717 | 0.864 |  |
|  | 6 | -111228 | 224766 | 223949 | 222971 | 0.866 |  |
| Year-4 visit (T2) (n=7920) | 1 | -131956 | 264289 | 264156 | 263996 | NA |  |
|  | 2 | -123426 | 247615 | 247345 | 247022 | 0.856 |  |
|  | 3 | -118242 | 237633 | 237226 | 236740 | 0.871 |  |
|  | 4 | -115641 | 232817 | 232274 | 231624 | **0.889** |  |
|  | 5 | -114447 | 230816 | 230136 | 229323 | 0.873 |  |
|  | 6 | -113348 | 229004 | 228188 | 227211 | 0.871 |  |
| Year-5 visit (T3) (n=7891) | 1 | -134229 | 268836 | 268703 | 268543 | NA |  |
|  | 2 | -124599 | 249962 | 249691 | 249369 | 0.924 |  |
|  | 3 | -120104 | 241357 | 240951 | 240465 | **0.873** |  |
|  | 4 | -117998 | 237531 | 236988 | 236339 | 0.886 |  |
|  | 5 | -116630 | 235181 | 234501 | 233689 | 0.879 |  |
|  | 6 | -115501 | 233309 | 232492 | 231516 | 0.868 |  |
| Note. LL = Log-Likelihood, CAIC = Consistent Akaike Information Criteria, BIC = Bayesian Information Criterion, SABIC = Sample-Size Adjusted Bayesian Information Criterion | | | | | | |  |
|  |  |  |  |  |  |  |  |
