# Supplementary material for: Longitudinal patterns of digital parenting restrictions and adolescent screen use: Insights from the Adolescent Brain Cognitive Development (ABCD) study
Source: PLOS Digit Health. 2026 Oct 28;5(10):e0001127. doi: 10.1371/journal.pdig.0001127 (PMC13716224; doi:10.1371/journal.pdig.0001127)
Supplement: Supplementary file 6 [file pdig.0001127.s006.docx]

| **S6 Table.** Fit Indices for Latent Transition Analysis Models With and Without Parameter Restrictions | | | | | | | |
| --- | --- | --- | --- | --- | --- | --- | --- |
|  | **# of parameters** | **LL** | **CAIC** | **BIC** | **SS-ABIC** | **Entropy** | **Diagonal Average Latent Class Probabilities** |
| Model with equality constraints | 140 | -361784 | 723848 | 724832 | 724388 | 0.873 | 0.917, 0.949, 0.933 |
| Model without equality constraints | 3416 | -326152 | 659136 | 683042 | 672186 | 0.93 | 0.938, 0.929, 0.944 |
| **Note.** The model without equality constraints failed to converge due to numerical instability, leading to potential local maxima and unreliable results. | | | | | | | |
| LL = Log Likelihood, CAIC = Consistent Akaike Information Criterion, BIC = Bayesian Information Criterion, SS-ABIC = Sample Size Adjusted Bayesian Information Criterion | | | | | | | |
