# Supplementary material for: Longitudinal patterns of digital parenting restrictions and adolescent screen use: Insights from the Adolescent Brain Cognitive Development (ABCD) study
Source: PLOS Digit Health. 2026 Oct 28;5(10):e0001127. doi: 10.1371/journal.pdig.0001127 (PMC13716224; doi:10.1371/journal.pdig.0001127)
Supplement: Supplementary file 7 [file pdig.0001127.s007.docx]

| **S7 Table.** Frequency of Parent Responses to the ABCD Parent Screen Time Questionnaire (Years 3-5) | | | | |
| --- | --- | --- | --- | --- |
|  | **Strongly Disagree** | **Somewhat Disagree** | **Somewhat Agree** | **Strongly Agree** |
| 1. When I am with my child, I use a screen-based device. | 2376 (10%) | 3557 (15%) | **11949 (50.5%)** | 5793 (24.5%) |
| 2. I try to limit how much I use a screen-based device when I am with my child | 1507 (6.4%) | 2090 (8.8%) | 9929 (42%) | **10136 (42.8%)** |
| 3. Our family often watches a screen during meals | **10689 (45.1%)** | 4462 (18.8%) | 5447 (23%) | 3117 (13.1%) |
| 4. Family members are allowed to use screen-based devices during meals | **12477 (52.6%)** | 4690 (19.8%) | 4568 (19.3%) | 1977 (8.3%) |
| 5. My child falls asleep while using a screen-based device | **13445 (56.7%)** | 3829 (16.2%) | 4527 (19.1%) | 1899 (8.0%) |
| 6. A screen-based device is usually playing in the room when my child falls asleep | **14351 (60.6%)** | 3167 (13.4%) | 3986 (16.8%) | 2190 (9.2%) |
| 7. My child has access to a mobile screen-based device in bed | **8596 (36.3%)** | 2291 (9.7%) | 5968 (25.2%) | 6829 (28.8%) |
| 8. I offer screen time to my child as a reward for good behavior | **13062 (46.1%)** | 3681 (16.0%) | 5013 (27.2%) | 1841 (10.6%) |
| 9. I take away screen time from my child as a punishment for bad behavior | 4881 (20.7%) | 2081 (8.8%) | 7129 (30.3%) | **9448 (40.1%)** |
| 10. I keep track of my child's screen time during the week | 5226 (22.1%) | 4693 (19.9%) | **8217 (34.8%)** | 5479 (23.2%) |
| 11. I keep track of my child's screen time during the weekend | 6174 (26.1%) | 5680 (24.0%) | **7453 (31.5%)** | 4336 (18.3%) |
| 12. I limit my child's screen time during the week. | 4489 (19.0%) | 3640 (15.4%) | 8311 (35.1%) | **7215 (30.5%)** |
| 13. I limit my child's screen time during the weekend. | 6013 (25.4%) | 6331 (26.8%) | **7235 (30.6%)** | 4080 (17.2%) |
| 14. I encourage my child to do activities other than screen time. | 797 (3.4%) | 387 (1.6%) | 3997 (17.0%) | **18277 (77.9%)** |
| Note. Frequencies represent combined responses across years. Percentages represent proportion of response type by item. | | | | |
