# Supplementary material for: Longitudinal patterns of digital parenting restrictions and adolescent screen use: Insights from the Adolescent Brain Cognitive Development (ABCD) study
Source: PLOS Digit Health. 2026 Oct 28;5(10):e0001127. doi: 10.1371/journal.pdig.0001127 (PMC13716224; doi:10.1371/journal.pdig.0001127)
Supplement: Supplementary file 8 [file pdig.0001127.s008.docx]

| **S8 Table.** Conditional Probabilities of Class Membership at Year 5 Based on Year 3 and 4 | | | |
| --- | --- | --- | --- |
| **Year 3 → Year 4** | **Probability of Low Restriction at Year 5** | **Probability of Moderate Restriction at Year 5** | **Probability of High Restriction at Year 5** |
| Low → Low Restriction | **0.793** | 0.174 | 0.032 |
| Low → Moderate Restriction | 0.323 | **0.638** | 0.039 |
| Low → High Restriction | **0.101** | 0.26 | 0.639 |
| Moderate → Low Restriction | **0.793** | 0.174 | 0.032 |
| Moderate → Moderate Restriction | 0.323 | **0.638** | 0.039 |
| Moderate → High Restriction | 0.101 | 0.26 | **0.639** |
| High → Low Restriction | **0.793** | 0.174 | 0.032 |
| High → Moderate Restriction | 0.323 | **0.638** | 0.039 |
| High → High Restriction | 0.101 | 0.26 | **0.639** |
| **Note:** The table presents the conditional probabilities of class membership at Year 5 given transitions from Year 3 to Year 4. Cells with higher probabilities indicate the most likely class progression. | | | |
