# Supplementary material for: Longitudinal patterns of digital parenting restrictions and adolescent screen use: Insights from the Adolescent Brain Cognitive Development (ABCD) study
Source: PLOS Digit Health. 2026 Oct 28;5(10):e0001127. doi: 10.1371/journal.pdig.0001127 (PMC13716224; doi:10.1371/journal.pdig.0001127)
Supplement: Supplementary file 9 [file pdig.0001127.s009.docx]

| **S9 Table.** Model Performance Metrics for Training and Test Sets | | |
| --- | --- | --- |
| **Metric** | **Training Set** | **Test Set** |
| R² | 0.146 | 0.102 |
| Root Mean Squared Error | 172.7 | 176.9 |
| Mean Absolute Error | 124.5 | 128.4 |
| **Note:** R² = The proportion of variance explained by the model | |  |
